# Coral reef soundscapes may not be detectable far from the reef

**DOI:** 10.1038/srep31862

**Published:** 2016-08-23

**Authors:** Maxwell B. Kaplan, T. Aran Mooney

**Affiliations:** 1 Biology Department, Woods Hole Oceanographic Institution, 266 Woods Hole Rd, MS50, Woods Hole, MA, 02543, USA; 2 Massachusetts Institute of Technology-Woods Hole Oceanographic Institution Joint Program in Oceanography/Applied Ocean Science and Engineering, Cambridge, MA, 02139, USA

## Abstract

Biological sounds produced on coral reefs may provide settlement cues to marine larvae. Sound fields are composed of pressure and particle motion, which is the back and forth movement of acoustic particles. Particle motion (i.e., not pressure) is the relevant acoustic stimulus for many, if not most, marine animals. However, there have been no field measurements of reef particle motion. To address this deficiency, both pressure and particle motion were recorded at a range of distances from one Hawaiian coral reef at dawn and mid-morning on three separate days. Sound pressure attenuated with distance from the reef at dawn. Similar trends were apparent for particle velocity but with considerable variability. In general, average sound levels were low and perhaps too faint to be used as an orientation cue except very close to the reef. However, individual transient sounds that exceeded the mean values, sometimes by up to an order of magnitude, might be detectable far from the reef, depending on the hearing abilities of the larva. If sound is not being used as a long-range cue, it might still be useful for habitat selection or other biological activities within a reef.

Coral reefs are hubs of marine biodiversity, and recruitment to these reefs provides the foundation of these ecosystems. Larval animals seeking the reef for settlement may use a variety of cues^1^. A range of sounds are produced on reefs, and it is increasingly realized that these acoustic components may assist pelagic larvae in locating and orienting toward suitable juvenile and adult habitat^2^,^3^,^4^,^5^. For example, experimental moorings that broadcasted reef sound attracted more fish larvae than silent controls^2^. Similarly, some juvenile fish species preferentially selected patch reefs broadcasting reef sound^5^. Reef sound has also been shown to induce metamorphosis in preparation for settlement in some crab larvae^6^.

It has been suggested that these reef soundscapes may offer a reef orientation cue at relatively large distances compared to some other stimuli^1^,^7^. Loud choruses of reef sounds may be detectable from hundreds to thousands of meters from the reef 8,9,10. A study by Egner and Mann^11^ proposed that sergeant major damselfish (*Abudefduf saxatilis*) would likely be able to use coral reef sounds as a significant navigation cue up to 500 m away. For some crab species, orientation and settlement responses were estimated at up to 40 km^12^. However, there are few studies quantifying the range over which reef sounds may actually propagate. This paucity of data limits our understanding of the extent to which this cue could be used by settling larvae. Furthermore, many sounds on the reef, such as fish choruses or snapping shrimp snaps, are produced with temporal periodicity^13^,^14^. This variation in sound levels suggests that the distances over which reef sound is detectable may also vary with time.

The ability of a given larva to detect sound cues will depend on its hearing abilities. While hearing has been studied in only a few of the approximately 12,000 extant marine fish species that live in shallow water or close to shore, and for whom acoustic settlement cues might be relevant^15^,^16^, those that have been studied generally show best sensitivities to low-frequency sound^15^. For example, juvenile sergeant major damselfish (*A. saxatilis*) were most sensitive to 100–400 Hz sounds in a hearing test^11^. No hearing studies have been carried out on larval marine invertebrates. Audiometric studies on adults are also somewhat limited, but like most fish, those tested (e.g., cephalopods and crustaceans) show lowest (most sensitive) thresholds below 1000 Hz^17^,^18^,^19^,^20^,^21^. Thus, it is critical to measure the propagation of reef sound at a range of frequencies, and particularly those low-frequency sounds that are within the hearing ranges of many marine fishes and invertebrates.

Sound is a mechanical disturbance that can be described in terms of its pressure or particle motion (i.e., velocity or acceleration)^22^. Most fish use their otolith system to detect the back-and-forth, vibratory-like, particle motion component of sound, whereas those with a swimbladder close to or connected to the ear can also detect sound pressure via auditory specializations^23^. Auditory physiological and morphological investigations of invertebrates indicate that those tested appear to be almost exclusively sensitive to particle motion (i.e., not pressure)^18^,^21^,^24^.

Bioacoustic larval settlement studies^2^,^3^,^5^,^12^,^25^ and investigations of the distances over which reef sound cues may travel have measured those cues in terms of pressure^8^,^26^, leaving responses to particle motion poorly understood. For example, Radford *et al*.^8^ modeled a reef as an extended sound source and found that reef sound pressure propagates much further than predicted by a point source. However, without acoustic particle motion data, the sound cues available to settling larvae remain largely unknown. Furthermore, the relationship between sound pressure and particle motion is not necessarily predictable close to the source in the near field^27^,^28^. Accordingly, to understand both pressure and particle motion cues, direct measurements are required. In addition, particle motion is inherently directional and thus can be used to identify the axis of a sound source, which is a vital attribute for closely spaced, low-frequency “ears” such as fish otoliths and invertebrate statoliths.

Indeed, there appear to be no direct measurements of reef particle motion in field conditions, limiting any estimation of the actual levels and ranges over which these settlement signals might be detectable. Nor are there data which quantify how this signal might vary in time. To address this deficiency, we carried out acoustic recording transects measuring both the particle motion and pressure components of reef sound at multiple distances from the reef. Measurements were made at two times of day (dawn and mid-morning) and repeated on three separate days to determine how the reef soundscape might change with time of day. Measurements were made in the summer (July 2015), coinciding with periods of fish and coral settlement^29^,^30^.

## Results

**Figure 1:**
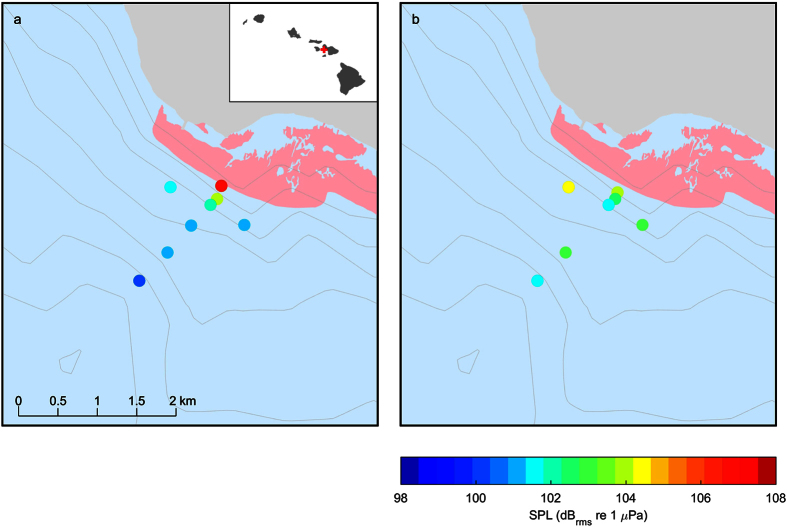
Map of Olowalu reef and transect point sound pressure levels (200–4900 Hz) at different distances from the reef during the (a) dawn and (b) mid-morning transects. Sound levels decreased with distance from the reef during the dawn transect but such a trend was not as obvious for the mid-morning transect. The pink shaded area denotes coral cover and the contour lines depict 10 m increments. The map data were obtained from NOAA National Centers for Environmental Information (http://maps.ngdc.noaa.gov/viewers/wcs-client/) and the State of Hawaii Office of Planning (http://planning.hawaii.gov/gis/download-gis-data/) and the figure was produced in Matlab 8.6 (The Mathworks, Natick, MA; http://www.mathworks.com/).

**Figure 2:**
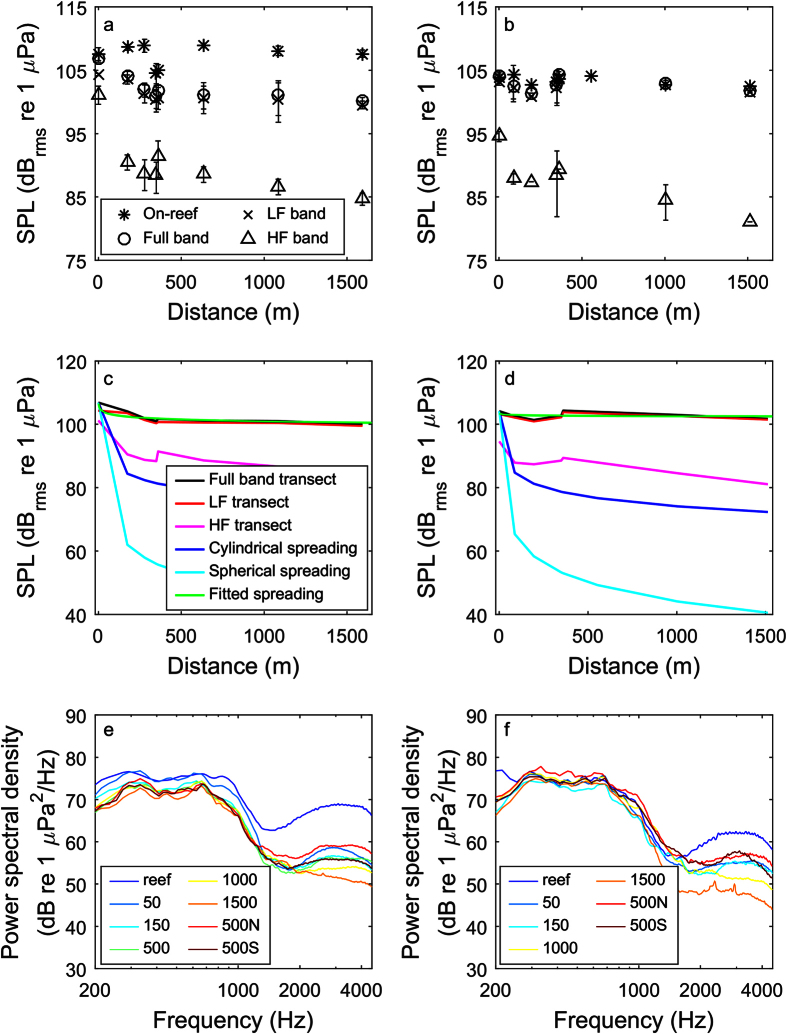
Mean (+/− SD) sound pressure levels (200–4900 Hz) on the reef (full band) and low- (200–1000 Hz), high-frequency (2000–4000 Hz) and full-band SPL at various distances from the reef at dawn (**a**) and mid-morning (**b**). Transect levels for the full band, low- (LF) and high-frequency (HF) shown with estimated cylindrical and spherical spreading loss and full band fit lines (**c,d**) and associated mean power spectra for each distance from the reef at the two times of day (**e,f**). On-reef sound levels stayed relatively constant during the dawn transect and varied over time during the mid-morning transect; conversely, full-band transect levels decreased with distance from the reef during the dawn but not the mid-morning transect. While high-frequency SPL attenuated with distance from the reef at both times of day, levels were considerably lower than the low-frequency SPL and accordingly make less of a contribution to the full-band trends. In neither case did attenuation match predicted spreading loss. High frequencies were elevated on the reef compared to every other transect point and decreased rapidly with distance from the reef. Low frequencies during the dawn transect appeared to decrease with distance from the reef while no such trend was evident for the mid-morning transect. No data were retained for the 500 m position during the mid-morning transect. See Table 2 for the equations of the fit lines in (**c,d**).

Sound pressure levels were generally highest on the reef and attenuated with distance during the dawn transect; such attenuation was not detectable during the mid-morning transect (Fig. 1; Table 1). On-reef levels stayed relatively constant over the course of the dawn transect, with one exception (c. 400 m, Fig. 2a) where levels were lower during the parallel transect. Full-band and low-frequency transect levels at dawn appeared to decrease along the transect with distance from the reef (Fig. 2a). On-reef levels were more variable during the mid-morning and decreased over the course of the transect (Fig. 2b). The mid-morning full-band transect levels were more variable than those at dawn and did not appear to decrease with distance from the reef. High-frequency sound pressure levels (SPL) did decrease with distance from the reef in both transects; however, because the levels were much lower than the low-frequency SPL, their contribution to the overall trend was limited. Indeed, low-frequency SPL tracked the full-band levels very closely as the low-frequency components contributed the majority of the energy to the full band. Attenuation of both the dawn and mid-morning transect sound level measurements was lower (more gradual) than was predicted by standard spherical or cylindrical spreading (Fig. 2c,d).

Power spectra followed a roughly similar shape across transect positions and time of day, with elevated low frequencies (less than 1000 Hz; Fig. 2e,f). These low frequencies attenuated with distance from the reef during the dawn transect. Such attenuation was again not apparent in the mid-morning transect, reflecting the fact that sound levels were lower. On-reef positions had elevated higher-frequency levels (2–4 kHz); during the dawn transect, sound levels were approximately 10 dB greater on the reef than the next closest position, while during the mid-morning on-reef levels were elevated but less so. These high-frequency, snapping shrimp components attenuated rather quickly immediately off the reef and then less so with increasing distance, which was expected given that attenuation rates increase with frequency. The position with the lowest high-frequency levels was the one furthest from the reef (1500 m distance).

**Figure 3:**
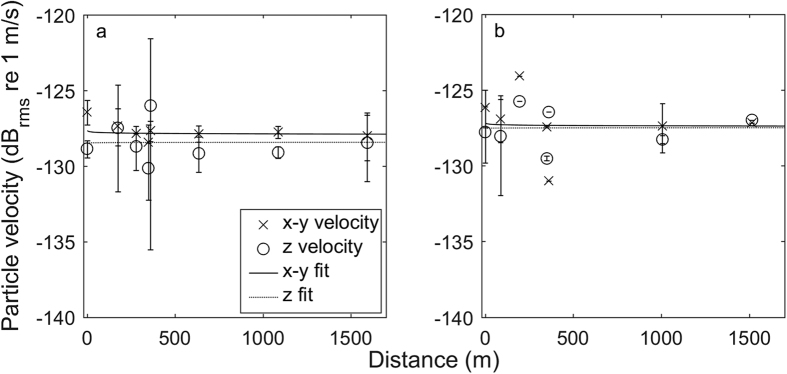
Mean (+/− SD) particle velocities for the horizontal (x-y) and vertical (z) axes of the M20 at a range of distances from the reef. Velocities in both major axes appeared to decrease with distance from the reef during the dawn (**a**) transect and in the x-y axes during the mid-morning (**b**) transect, but there was considerable variability. Vertical velocities were nearly as high as horizontal velocities for both transects. No data were available for the 500 m position during the mid-morning transect. See Table 2 for equations of the fit lines.

Particle velocities in the horizontal (x-y) and vertical (z) axes appeared to decrease with distance from the reef during the dawn transect and in the horizontal axis only during the mid-morning transect, although there was considerable variability around the mean (Fig. 3; Table 1). Notably, the vertical velocities were roughly the same magnitude as the horizontal velocities in both transects.

Empirically derived spreading relationships for sound pressure and particle velocity in the horizontal and vertical axes were only statistically significant during the dawn transect for sound pressure and velocity in the x-y axis (Table 2).

**Figure 4:**
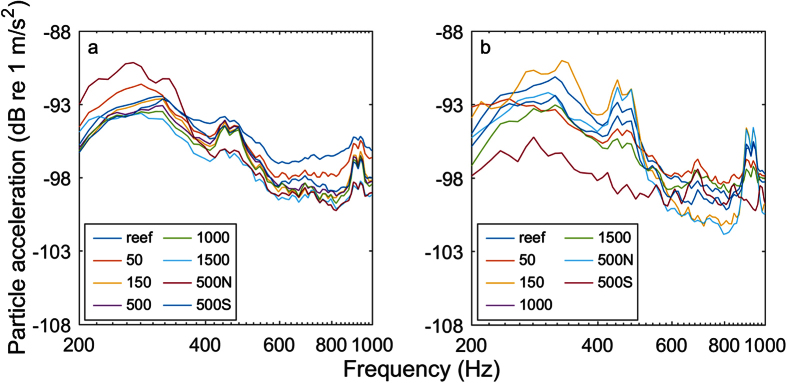
Mean particle acceleration values by transect, position, and frequency in the x-axis. Peaks in acceleration were seen below 500 Hz in both transects, but no clear pattern with distance was evident. Acceleration was elevated slightly at the 500 N position during the dawn transect (**a**) and at the 150 m position during the mid-morning transect (**b**). No data were available for the 500 m position during the mid-morning transect.

Acceleration values in the x-axis were elevated at frequencies below 500 Hz in the dawn and mid-morning transects but did not follow a clear pattern with distance from the reef (Fig. 4). Similar patterns were seen in the y and z axes (not shown). The transect position with the highest low-frequency acceleration values was the 500 N position during the dawn transect and the 150 m position during the mid-morning transect, although in both cases levels were similar across positions and times of day.

**Figure 5:**
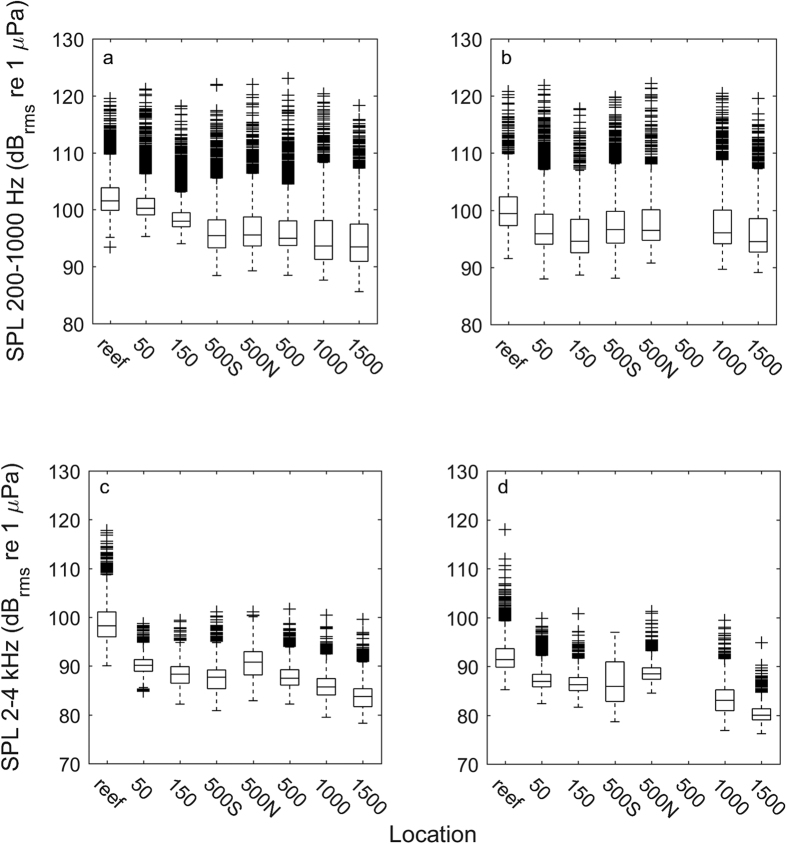
Boxplots showing SPL in 5 ms windows in low- (200–1000 Hz) and high-frequency (2000–4000 Hz) bands ((a–d) respectively) during the dawn (a,c) and mid-morning (b,d) transects pooled across sampling days. While the median SPL values roughly follow the same trend as that for the mean values displayed in Fig. 3, there is considerable spread (in particular in the low-frequency band) and there are multiple high-amplitude outliers for nearly every recording position. The number of high-amplitude outliers relative to the lower amplitude averages suggests that while larval animals may not be able to hear the average sound from a reef, it is possible that they could detect some high-amplitude transients. No data were available for the 500 m position during the mid-morning transect. Central bar – median; box – 25–75 percentiles; whiskers – most extreme data points not considered as outliers; crosses – outliers.

Median sound levels based on 5 ms segments of each recording pooled across days showed a similar trend to the averages with respect to attenuation with distance (Fig. 5). However, there were a considerable number of high-amplitude outliers for both the dawn and mid-morning transect and for both the low- and high-frequency bands, although there were more outliers in the low-frequency band.

## Discussion

A range of studies have demonstrated that larval fish and invertebrates may orient towards reef sound and perhaps even discern among sound types; however, there are very limited data on the distances over which acoustic cues might be used. Furthermore, there have been few direct measurements of acoustic particle motion, the relevant acoustic cue for most larval fishes and invertebrates, thus leaving this potential settlement cue overlooked. The current investigation represents an effort to fill this knowledge gap by quantifying this particle motion cue, particularly with respect to the distances over which reef sound propagates.

Reef sound pressure did attenuate with distance from the reef but this varied both by frequency and time of day, likely as a result of variation in sound production. Attenuation was most apparent at dawn in both frequency bands and during the mid-morning in the high-frequency band only. Notably, the dip in sound level for the on-reef recorder (at the 400 m mark) during the dawn transect (Fig. 2b) may reflect some of this diel periodicity as this was a parallel transect point and thus the last (latest) taken during each recording session. Coral reef soundscapes have a strong temporal periodicity^13^,^14^. Given that sound production on many reefs including Hawaiian reefs has been shown to peak at dusk and dawn^31^, it is possible that these times of day represent a reliably higher sound level that larvae may use to orient. However, whether and how changing sound levels over time might affect larval sensing has yet to be determined.

The lack of agreement with cylindrical or spherical spreading predictions has been demonstrated on temperate reefs and has been postulated to be a result of a ”reef effect”^8^, whereby levels do not attenuate as quickly as predicted because the reef is not a single point source but instead is composed of a “chorus” of many sound sources (fish, snapping shrimp). However, aural and visual inspections of our coral reef recordings collected far from the reef indicated no discrete detectable sounds of biological origin, which suggests that at greater distances from the reef such sounds might be buried in the environmental noise floor. Alternatively, the temperate reefs, which are acoustically dominated by feeding urchins^8^, may simply exhibit a different propagation pattern from coral reefs rich in fish and snapping shrimp sounds. In addition, propagation may be largely dependent on bathymetry and substrate^32^.

Earlier work stressed the importance of identifying which coral reef soundscape components are the vital settlement cues: lower-frequency fish sounds or higher-frequency shrimp sounds^33^. The data presented here show that reef sound at high frequencies attenuates rapidly and that the particle acceleration and pressure levels in this band are relatively low. This suggests that it is unlikely that the high-frequency shrimp cues could be detected at any appreciable distance from the reef.

Here we show that the lower-frequency fish sounds were both higher in amplitude and propagated farther, particularly during crepuscular chorus events. In addition, there was a much larger number of high-amplitude transients in the low-frequency band. Furthermore, this frequency band overlaps the predominant hearing range of many of the marine fish and invertebrates that have been tested^15^,^18^, suggesting that both the low-frequency chorus and/or these transients may offer viable cues for settlement. While sound levels appeared to be somewhat elevated below 200 Hz compared to levels between 200–1000 Hz, the former frequency range was removed by filtering to ensure that the reported levels were predominately a result of biological sound production.

Particle velocity data collected on the M20 particle velocimeter corresponded somewhat to sound pressure data collected on the DMON acoustic recorder in terms of attenuation with distance, but there was considerable variability. The z axis showed velocities that were roughly as high as in the x-y axes. The reason for this is not obvious; while it may be a result of the influence of non-acoustic sources (i.e., up-down motion from surface waves on the mooring system) despite the multiple absorption methods, physical movements of the mooring system are expected to be much lower frequency than the analysis bandwidths used here.

The acceleration data did not display any clear trends with distance from the reef in either transect. Furthermore, the position with the highest acceleration values was different in the dawn transect (500 N) than in the mid-morning transect (150 m). This differs from the velocity data (which do show a trend with distance). This is perhaps due to the fact that the acceleration data are frequency-specific; as a result, these data are more variable than the velocity data to which they are proportional.

Given that many fishes are likely able to sense particle motion and that it is the primarily hearing modality for larval fishes^9^ it is instructive to compare the particle acceleration values measured here to hearing thresholds. Radford, *et al*.^34^ measured particle acceleration-derived hearing thresholds using a shaker table for the goldfish (*Carassius auratus*), bigeye (*Pempheris adspersa*), and common triplefin (*Forstergyian lappillum*). The best hearing sensitivity for all three species was between 100–200 Hz and was approximately −60 dB re 1 m/s^2^, which is approximately 25 dB higher than the highest acceleration values measured at any position in this study, indicating that those species probably could not hear the average Olowalu reef signal at any of these distances. Conversely, Horodysky, *et al*.^35^ measured the hearing thresholds using a speaker in air for six sciaenid species and found considerably lower particle acceleration thresholds (approximately −100 dB re 1 m/s^2^ for the Atlantic croaker at 100 Hz), which suggests that some fishes could detect this reef at the distances measured. While the hearing abilities of larval and the majority of adult invertebrates have yet to be measured, Mooney, *et al*.^18^ measured the acceleration-based hearing thresholds of adult longfin squid (*Doryteuthis pealeii*) and found that their best thresholds were −35 dB re 1 m/s^2^ at 200 Hz, indicating that these squid could not hear this reef at any distance. Similarly, Packard, *et al*.^24^ tested the low-frequency hearing of cuttlefish (*Sepia officinalis*), squid (*Loligo vulgaris*), and octopus (*Octopus vulgaris*) and identified a threshold level of −40 dB re 1 m/s^2^ at 10 Hz. Finally, Roberts, *et al*.^36^ tested the response to vibration of the blue mussel (*Mytilus edulis*) and found a best sensitivity of −22 dB re 1 m/s^2^ at 10 Hz. Because reef sound is broadband and these hearing thresholds are derived using pure tones, it is prudent to increase the audiogram sensitivity by 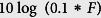, where F is the tone frequency, to account for the critical bandwidth^11^. However, doing so does not change which of these species could theoretically detect the reef based on the available data.

The majority of these hearing studies did not report background noise levels (but see^36^). The amount that individual signals are elevated with respect to the background noise is critical to determining both whether a larva could detect the signals and the potential for masking. For example, a study on the hearing abilities of goldfish (*C. auratus*), catfish (*Platydoras costatus*), and sunfish (*Lepomis gibbosus*) found that in the presence of masking noise hearing thresholds are considerably elevated^37^.

The reef sound levels reported here are averages over a minute of recording. Thus, there will be variability above and below the mean, with some sounds exceeding the average levels. Whether those sounds are detectable will depend on their amplitude, spectral composition, the amount by which they exceed the background noise, and the larval hearing abilities of a given species. Indeed, when the SPL of short (5 ms) segments was pooled by recording position across the recording days and transect times, there were a considerable number of high-amplitude transient signals, some of which were 20 dB (i.e., an order of magnitude) above median levels (Fig. 5). Many more of these transients were found in the low-frequency band, where larval hearing is likely more sensitive, than in the high-frequency band. Accordingly, while larval animals might not be able to hear the average sound level at any appreciable distance from the reef, they might detect some of these transient signals. Indeed, it is possible that an individual high-amplitude signal that originated on the reef might propagate according to theoretical predictions; however, detection of such cues was not possible in the present study design.

In conclusion, coral reef sound pressure levels attenuated with distance from the reef but not to the extent predicted by theory, perhaps as a result of the ambient noise levels of the area and the relatively shallow water (less than 100 m) off the reef. Attenuation with distance from the reef was more evident during dawn when, crepuscular biological activity is greatest. By mid-morning, such a relationship between sound level and distance was not apparent. Similar trends were apparent for particle velocity but with considerable variability, and no such trend was apparent for particle acceleration. Because there was no particle velocity sensor on the reef for the duration of each transect, it is not possible to state unequivocally that on-reef particle velocity levels were relatively constant over the course of the transect, even though within-transect temporal variability in sound pressure levels was limited. Average particle acceleration levels were at or below published hearing thresholds, which suggests that these sounds may be perhaps too faint to be used as an orientation cue for marine larvae in search of suitable settlement habitat, at least at distances beyond 150 m. However, the high amplitude transient signals present at nearly every distance from the reef and on both transects might be detectable at greater ranges. Additionally, if other reefs or other times of day exceeded the mean values presented here, those might be detectable, depending on their attributes and the hearing abilities of a given larva.

These data show the temporal and spatial scales of the available acoustic cues vary, yet seemingly do not offer a long-distance (several km) cue. However, the actual scales over which acoustic cues may function, at particular times and places, remain unresolved. If larvae are not using sound as a long-distance orientation cue but are responding directionally to sound, perhaps acoustic cues could offer a shorter range cue for settling animals; for example, once they are close to a reef. As many larvae approach reefs at night (when vision is presumably limited) sound cues might offer an indication that the larva is above that reef and could then successfully settle. More comprehensive measurements at key times (i.e., night) would be valuable to assess the scales over which reef sounds may play a role in larval processes. Additionally, acoustic signals are now known to operate as a contact call for some fishes^38^. Sound cues might thus reflect finer-scale assessments of a reef such as habitat of nearby soniferous predators or conspecifics to respectively avoid or approach.

## Methods

This study was conducted on July 17, 19, and 20, 2015 immediately after the new moon at one southwest-facing reef, Olowalu, which is located on Maui’s (HI, USA) leeward side (N 20.802, W156.618). During the data collection period, sea state on all three of these days was less than Beaufort 2 (wind 4–6 knots, wave heights 0.2–0.5 m). Olowalu is a broad, relatively flat reef dominated by branching corals with some massive corals and sand channels. Much of the reef is found from approximately 6–11 m depth. This reef has approximately 70% live coral cover, and 33 fish species were observed in visual surveys (unpublished data, February 2015). The benthic cover changes from coral-dominated structure to sand with increasing distance from the reef.

**Figure 6:**
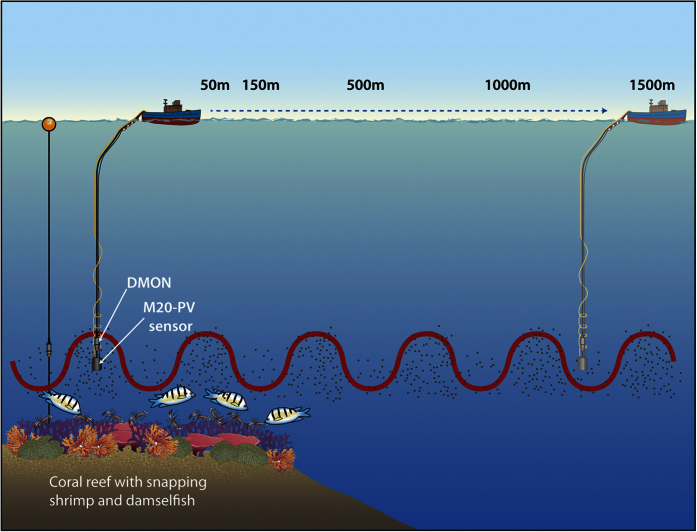
Schematic of the data collection transect. Acoustic recordings were collected on the reef with a DMON affixed to the mooring on the left and from the vessel using both a DMON and the M20 particle velocity sensor. This figure is not covered by the CC BY license (Image courtesy of Jack Cook, WHOI Graphic Services). All rights reserved, used with permission.

Acoustic data were collected as follows. At the start of the experiment, a stationary DMON digital acoustic recorder (Woods Hole Oceanographic Institution, Woods Hole, MA, USA) was deployed on a mooring on the reef (approximately 1 m off the bottom) to record sounds directly on the reef and monitor the natural temporal changes in sound production during the course of the study (Fig. 6). The mooring was deployed at the same location as another acoustic recording device, which has been collecting data at this position for approximately 18 months as part of a different study. This mooring DMON recorded using a low-frequency hydrophone element (Navy type II ceramic) with a high-pass filter at 8 Hz and 60 kHz sample rate (down-sampled to 10 kHz before commitment to onboard FLASH memory).

To measure the reef sound propagation, vessel-based transects were conducted, recording reef sounds at various distances from the reef. The vessel recording package consisted of two instruments: a DMON set up as above to measure sound pressure in a manner identical to the mooring, and a M20 particle velocimeter (GeoSpectrum Technologies Inc, Dartmouth, NS) to record particle velocity (i.e., the velocity component of acoustic particle motion). The M20 contains three vector sensors (i.e., accelerometers), an omnidirectional hydrophone, and digital magnetometer/accelerometer chip for instrument orientation sensing housed in a negatively buoyant aluminum can. The M20 has a usable frequency range of up to 3 kHz; accordingly, sample rates of either 6000 or 12500 Hz were used for all four channels, with data recorded directly onto a laptop via a data acquisition card (USB-6002; National Instruments Corporation, Austin, TX), using a customized Matlab (The Mathworks, Natick, MA) program. The instruments were deployed at approximately 10 m depth (the maximum possible depth given the depth of the reef) on a mooring consisting of two communication cables married to a ¼-inch polypropylene line. An Anchor Buddy (Greenfield Products, Greenfield, OH) was fastened to the top and bottom of the mooring setup to dampen wave-driven vertical movements. The M20 was affixed by eye bolt to the bottom of this line with the DMON slightly above it, oriented in a hydrophone-down position. A 2-lb lead weight was affixed to the line adjacent to the DMON (i.e., above the M20) to help ensure a vertical mooring orientation. At the surface, 12 1-lb gillnet floats were associated to the line in an effort to further dampen wave activity.

Recordings were made at specific points (Fig. 1) along two transects, one perpendicular and one parallel to the reef at two times of day – dawn and mid-morning. First, the perpendicular transect from the reef out to approximately 1500 m was carried out, followed by the parallel transect. On the perpendicular transect, at minimum recordings were made on the reef and at 50, 150, 500, 1000 and 1500 m. On July 17 and July 20 some other locations were sampled, but those were excluded from subsequent analyses because of the lack of multiple days of recording at those positions (Table 3). On the parallel transect, two points were selected, one north and one south of the perpendicular transect. These points were both parallel with the 500 m position of the perpendicular transect but were 360 and 349 m away respectively from the projected tip of the reef (based on map data; see Fig. 1). Recording duration at each point was 2.5 min and the recordings were made with the vessel engine off to minimize acoustic and physical disturbance to the acoustic recording instruments. Each set of transects took approximately 60 min to complete (except for the first dawn transect during which several additional recordings were made). Water depth along the transect varied from approximately 11 m at the reef to approximately 60 m at the 1500 m position.

All acoustic analyses were carried out in Matlab 8.6. Each recording from both DMONs and the M20 was reduced into a 1-minute segment to remove any extraneous noise associated with deployment. Any segments containing vessel noise were excluded from subsequent analyses; however, segments with limited wave noise were retained. The remaining segments were detrended to remove any DC offset, corrected for instrument sensitivity, and band pass filtered at 200–4900 Hz (full band DMON), 200–1000 Hz (low-frequency (LF) band DMON), 2–4 kHz (high-frequency (HF) band DMON), and 200–2800 Hz (M20) using four-pole Butterworth filters. The high pass filter (200 Hz) for all bands was set to ensure that any low-frequency periodic signals from physical motion (e.g., surface waves) were significantly (i.e., orders of magnitude) below the filter’s frequency.

Sound pressure levels (SPL; root-mean-square) and power spectral density estimates (FFT size: 512; Hann window, 75% overlap) were calculated for the entire 1-minute segment of the DMON hydrophone recordings. In addition, SPL was also calculated in 5 ms windows in the DMON LF and HF band to examine variability within the minute-long records.

Mean particle velocity measurements and power spectral density estimates (same spectral settings as above) were calculated in the 200–2800 Hz band for each of the M20 accelerometer axes. As a result of the sensor orientation chip malfunction, it was not possible to correct the particle velocity axes with measured pitch, roll, and heading. Nevertheless, because the bottom of the mooring was weighted, the z axis can be reliably referred to as the vertical axis and the x- and y-axes as the horizontal axes. Accordingly, the x- and y-axes were combined so as to present a total magnitude of horizontal velocity.

Attenuation with distance from a source is a result of spreading loss and absorption, the latter of which is negligible at these frequencies and distances^39^. Spreading loss can be modeled as spherical (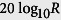) or cylindrical 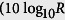) spreading, where 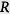 is range from the source in meters. These estimated losses were compared to the measured changes in sound pressure level with distance from the reef. To quantify the *in situ* spreading, exponential decay functions were fitted to the sound pressure and particle velocity attenuation data.

Particle acceleration, the temporal differentiation of particle velocity, can be found using the relationship 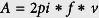, where A is the particle acceleration, f is frequency and v is particle velocity. This was done with each axis individually to compare measured values to published acceleration-based hearing thresholds of fishes and invertebrates. To focus on the diel variability in sound production and attenuation with distance from the reef, mean values across days for each transect position and time of day are presented with standard deviations (both calculated on the linear scale). Only positions on the transect that were recorded more than once are reported here.

## Additional Information

**How to cite this article**: Kaplan, M. B. and Mooney, T. A. Coral reef soundscapes may not be detectable far from the reef. *Sci. Rep.* **6**, 31862; doi: 10.1038/srep31862 (2016).

## Figures and Tables

**Table 1:** Mean sound pressure and particle velocity values by transect and position.

Position	Mooring DMON SPL		Transect DMON SPL		Transect M20 SPL		M20 PV X		M20 PV Y		M20 PV Z	
	Dawn	Mid-Morning	Dawn	Mid-Morning	Dawn	Mid-Morning	Dawn	Mid-Morning	Dawn	Mid-Morning	Dawn	Mid-Morning
Reef	107.6	103.7	106.8	104.1	122.0	117.3	−129.9	−129.6	−129.1	−128.7	−128.9	−127.8
50 m	108.6	104.2	104.1	102.5	115.8	113.5	−130.3	−130.1	−130.4	−129.8	−127.5	−128.1
150 m	108.9	102.6	102.4	101.4	115.4	114.3	−127.6	−125.2	−128.4	−130.4	−120.5	−125.8
500 m	108.9	104.0	101.2	—	116.0	—	−130.8	—	−131.0	—	−129.2	—
1000 m	108.1	102.6	101.0	103.0	115.7	112.7	−130.5	−129.8	−131.0	−131.2	−129.1	−128.3
1500 m	107.6	102.5	100.1	101.7	113.0	113.5	−131.4	−130.5	−130.7	−129.9	−128.5	−127.0
500 S m	104.6	103.1	101.0	102.8	114.5	113.7	−131.3	−130.6	−131.6	−130.4	−130.2	−129.5
500 N m	105.0	103.6	101.7	104.3	116.3	111.7	−130.0	−133.9	−131.5	−134.1	−126.0	−126.5

SPL in dB re 1 μPa and PV in dB re 1 m/s. DMON and M20 values are full band (200–4900 Hz and 200–2800 Hz, respectively).

**Table 2:** Exponential decay equations of the curve fit to full band sound pressure level (in dB re 1 μPa) and particle velocities (in dB re 1 m/s) displayed in Figs 3c,d and 4a,b respectively, where y is the sound level at a given distance and x is the distance from the reef.

Transect	SPL_rms_	SPL r^2^	SPL p-value	XY Velocity	XY r^2^	XY p-value	Z Velocity	Z r^2^	Z p-value
Dawn	y = 107.1*x^−0.0086^	0.889	0.001	y = −127.6*x^0.0003^	0.742	0.02	y = −128.5*x^−0.00007^	0.011	0.76
Mid-morning	y = 103.6*x^−0.0015^	0.105	0.50	y = −127*x^0.00023^	0.051	0.51	y = −127.6*x^−0.00005^	0.006	0.86

**Table 3:** Summary of point data collection combined among perpendicular and parallel transects.

Date	Start time (local)	End time (local)a	Number of points on transect	Number of points after boat noise exclusion
17/07/15	5:01	6:51	15	14
17/07/15	9:01	9:53	8	2
19/07/15	5:01	5:58	8	8
19/07/15	9:40	10:27	8	4
20/07/15	4:55	6:03	10	9
20/07/15	8:57	9:48	10	8
